# UV-A-induced structural and functional changes in human lens deamidated αB-crystallin

**Published:** 2008-02-01

**Authors:** Kerri Mafia, Ratna Gupta, Marion Kirk, L. Wilson, O.P. Srivastava, Stephen Barnes

**Affiliations:** 1 Departments of Pharmacology & Toxicology; 2 Vision Sciences; 3 The Comprehensive Cancer Center, University of Alabama at Birmingham, the Purdue University-University of Alabama at Birmingham Botanical Center for Age-Related Disease, Birmingham, AL

## Abstract

**Purpose:**

To determine comparative effects of ultraviolet (UV)-A irradiation on structural and functional properties of wild type (WT) αB-crystallin and its three deamidated mutant proteins (αB-Asn78Asp, αB-Asn146Asp, and αB-Asn78/146Asp).

**Methods:**

Three deamidated mutants previously generated from recombinant WT αB-crystallin, using a site-specific mutagenesis procedure as previously described [32], were used. The WT αB-crystallin and its three deamidated species were exposed to UV-A light (320–400 nm) at intensities of 20 or 50 J/cm^2^. The UV-A-unexposed and UV-A-exposed preparations were examined for their chaperone activity, and their activities were correlated with the UV-A-induced structural changes. The structural properties studied included dimerization and degradation, intrinsic tryptophan (Trp) fluorescence, ANS (8-anilino-1-naphthalenesulfate)-binding, far ultraviolet circular dichroism (UV-CD) spectral analysis, molecular sizes by dynamic light scattering, and oxidation of Trp and methionine (Met) residues.

**Results:**

The WT αB-crystallin and its three deamidated mutant proteins showed enhanced dimerization to 40 kDa species and partial degradation with increasing doses during UV-A-exposure. Compared to the deamidation of asparagines (Asn) 78 residue to aspartic acid (Asp) or both Asn78 and Asn146 residues to Asp, the deamidation of Asn146 residue to Asp resulted in a greater loss of chaperone activity. The UV-A-induced loss of chaperone activity due to structural changes was studied. The ANS-binding data suggested that the αB-Asn146Asp mutant protein had a relatively compact structure and an increase in surface hydrophobic patches compared to WT and two other deamidated proteins. Similarly, UV-A-exposure altered the Trp microenvironment in the deamidated mutant proteins compared to the WT αB-crystallin. Far-UV CD spectral analyses showed almost no changes among WT and deamidated species on UV-A-exposure except that the αB-Asn146Asp mutant protein showed maximum changes in the random coil structure relative to WT αB-crystallin and two other deamidated proteins. The UV-A-exposure also resulted in the aggregation of WT and the three deamidated mutant proteins with species of greater mass compared to the non-UV-A exposed species. Among the four spots recovered after two-dimensional (2D)-gel electrophoresis from WT and the three deamidated species, the Met and Trp residues of αB-Asn146Asp mutant showed maximum oxidation after UV-A exposure, which might account for its greater loss in chaperone activity compared to WT αB-crystallin and two other deamidated species.

**Conclusions:**

After UV-A-exposure, the deamidated αB-Asn146Asp mutant protein showed a complete loss of chaperone activity compared to WT αB and αB-Asn78Asp and αB-Asn78/146Asp deamidated species. Apparently, this loss of chaperone activity was due to oxidative changes leading to its greater structural alteration compared to other αB-species.

## Introduction

Lens structural proteins (α-, β-, and γ-crystallines) by virtue of their high concentration and unique interactions focus incoming light onto the retina and maintain lens transparency during the majority of our lifetime. Among the crystallines, α-crystallin is made of two subunits, αA (173 amino acid residues) and αB (175 amino acid residues), which apparently play a critical role in lens transparency because of their chaperone activity [1]. The αA- and αB-crystallines show approximately 55% sequence homology [2], are composed of the highest percentage of total lens proteins (35%) [3], exist as oligomers of approximately 800 kDa, and are members of the small heat shock protein (sHsp) superfamily [4-6].

αA-crystallin is lens specific. However, αB-crystallin, although present at a high concentration in the lens, is also found in other tissues, including brain, the lung, and cardiac and skeletal muscles [7]. Further, the expression of αB-crystallin is upregulated under stress such as the overexpression of αB-crystallin in the development of benign tumors associated with tuberous sclerosis, neuromuscular disorders [8], and other neurological diseases like Alexander’s, Alzheimer, and Parkinson diseases [8,9].

The stress on cells could be intrinsic such as oxidation, phosphorylation, and deamidation of proteins or extrinsic such as heat or UV irradiation. Ultraviolet (UV) irradiation is one of the stress factors that are believed to cause age-related cataract [10,11]. Sunlight consists of ultraviolet radiation, which is made up of UV-A (composed of longer wavelengths between 320 to 400 nm) and ultraviolet (UV)-B (composed of shorter wavelengths between 280 and 320 nm), and both have destructive properties that can cause cataract [10,11]. An association between cortical cataracts and UV-A radiation has been established [12]. The human lens absorbs all the impinging UV-A radiation between 320 to 400 nm because of intrinsic UV filters [13,14]. It is believed that a cortical cataract begins at the inferonasal lens [15,16] where the sunlight is most concentrated [17]. An epidemiological correlation between high levels of the UV component of sunlight to higher incidence of cataracts in humans has been established [18,19]. UV-A-induced oxidation of lens proteins [12,20], DNA [21], and membranes [22] has been shown as well as the formation of singlet oxygen (^1^O_2_) species [12].

Age-related cataract is believed to be a consequence of the aggregation of α-, β-, and γ-crystallines and the subsequent precipitation of the aggregates and cross-linked products. The precipitation of crystallines is thought to be initiated by posttranslational modifications, which change their structural and functional properties. Recent studies of water insoluble (WI) proteins from normal human lenses showed that crystallines undergo in vivo modifications, which included disulfide bonding, deamidation, oxidation, and backbone cleavage [23-26]. However, additional modifications (i.e., glycation [27], oxidation of tryptophan (W) and histidine (H) residues [28,29], deamidation [30-33], transglutaminase-mediated cross-linking [34], and racemization [35,36]) in crystallines are also believed to contribute to aggregation and cross-linking. Although the exact mechanism of UV-A-induced cataract with advancing age is not known, several pieces of evidence suggest that oxidation of specific amino acids such as Trp and Met might be the causative factor. How the oxidatively modified Met and Trp cause lens crystallins to aggregate and form cross-linked products is not known. Although the effects of UV-A-exposure on individual α-, β-, or γ-crystallins or on lens enzymes have been investigated in past studies [12,20,37], its effects on the posttranslationally modified crystallines have not been studied.

Deamidation of crystallines is a nonenzymatic process. David et al. [38] have reported that deamidation is the most prevalent modification compared to other modifications found in crystallines. Both Asn and glutamine (Gln) residues undergo deamidation, which results in the introduction of a negative charge at the site of modification and may alter the protein’s tertiary structure and affect its biologic properties. It is expected that deamidation changes the protein’s three-dimensional structure. Lampi [39] recently reported that the deamidation and not truncation in human βB1-crystallin reduced its stability. Also, we showed the presence of Asn146 deamidated species of αB-crystallin in the normal and cataractous human lenses [40]. Recently, we showed that the deamidation of Asn146 but not of Asn78 had a profound effect on the structural and functional properties of human αB-crystallin [31]. Similarly, our study showed that both Asp residues (Asp101 and Asp123) of human αA-crystallin are required for the structural integrity and chaperone function of the crystallin [41].

Although the UV-A-irradiation is believed to be a major initiating and causative factor for age-related cataract, the effects of the irradiation on deamidated crystallines are presently unknown. In the present study, we determined comparative effects of UV-A irradiation on wild type (WT) αB-crystallin and its three deamidated mutant proteins (αB-Asn78Asp, αB-Asn146Asp, and αB-Asn78/146Asp) on their structural and functional properties.

## Methods

### Materials

Molecular biology grade chemicals were purchased from either Sigma-Aldrich (St. Louis, MO) or Thermo-Fisher (Norcross, GA) unless stated otherwise. DEAE-Sephacel and Butyl-Sepharose were from GE Biosciences (Piscataway, NJ).

**Figure 1:**
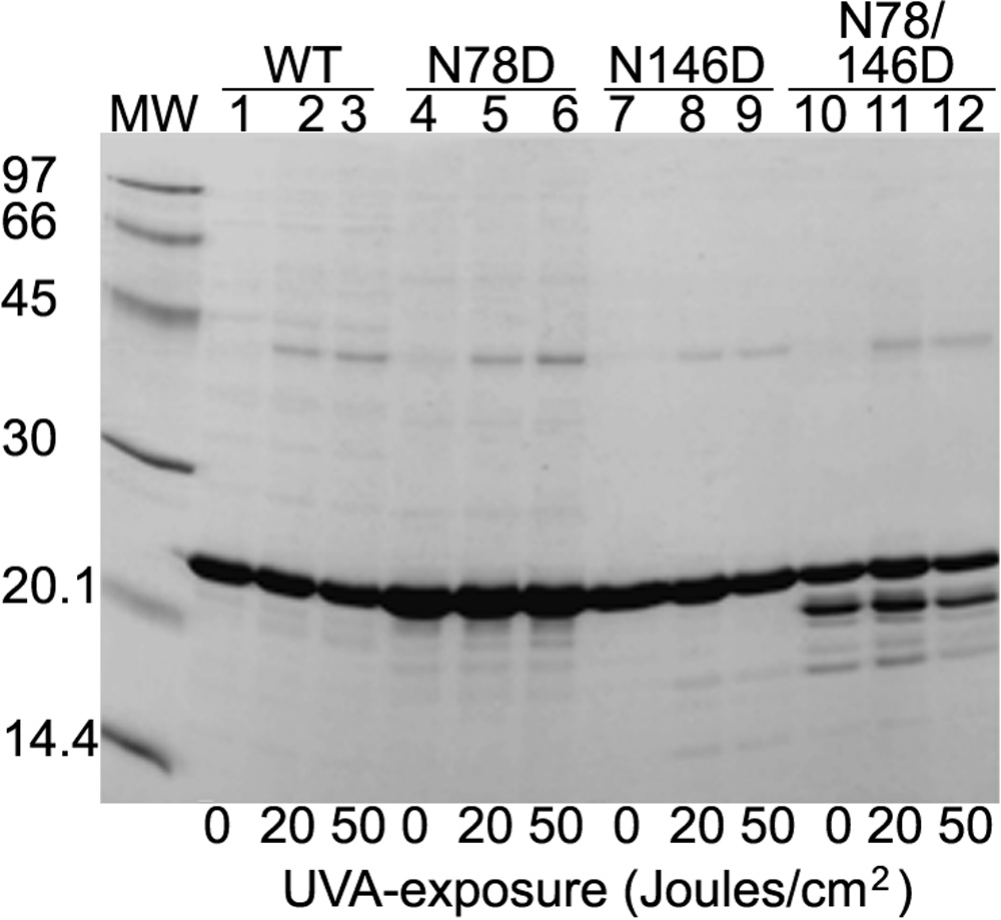
SDS–PAGE analysis of UV-A-exposed and unexposed WT αB-crystallin and its three deamidated mutant species. After UV-A-exposure of varying doses (0, 20, and 50 J/cm^2^, shown at the bottom of the gel), the WT αB-crystallin and its three deamidated mutant proteins (αB-Asn78Asp, αB-Asn146Asp, and αB-Asn78/146Asp) were analyzed. Increased dimerization of each protein and degradation, particularly in the deamidated species, were observed following UV-A-exposure.

### Bacterial strains and plasmids

*E. coli* BL21 (DE3), and *E. coli* BL21 (DE3) pLysS bacterial strains were obtained from Promega (Madison, WI). The human αB-crystallin cDNA cloned in plasmid pDIRECT was received from Dr. Mark Petrash of Washington University, St. Louis, MO. Cells were propagated in Luria broth, and recombinant bacteria were selected using ampicillin and chloramphenicol.

**Figure 2:**
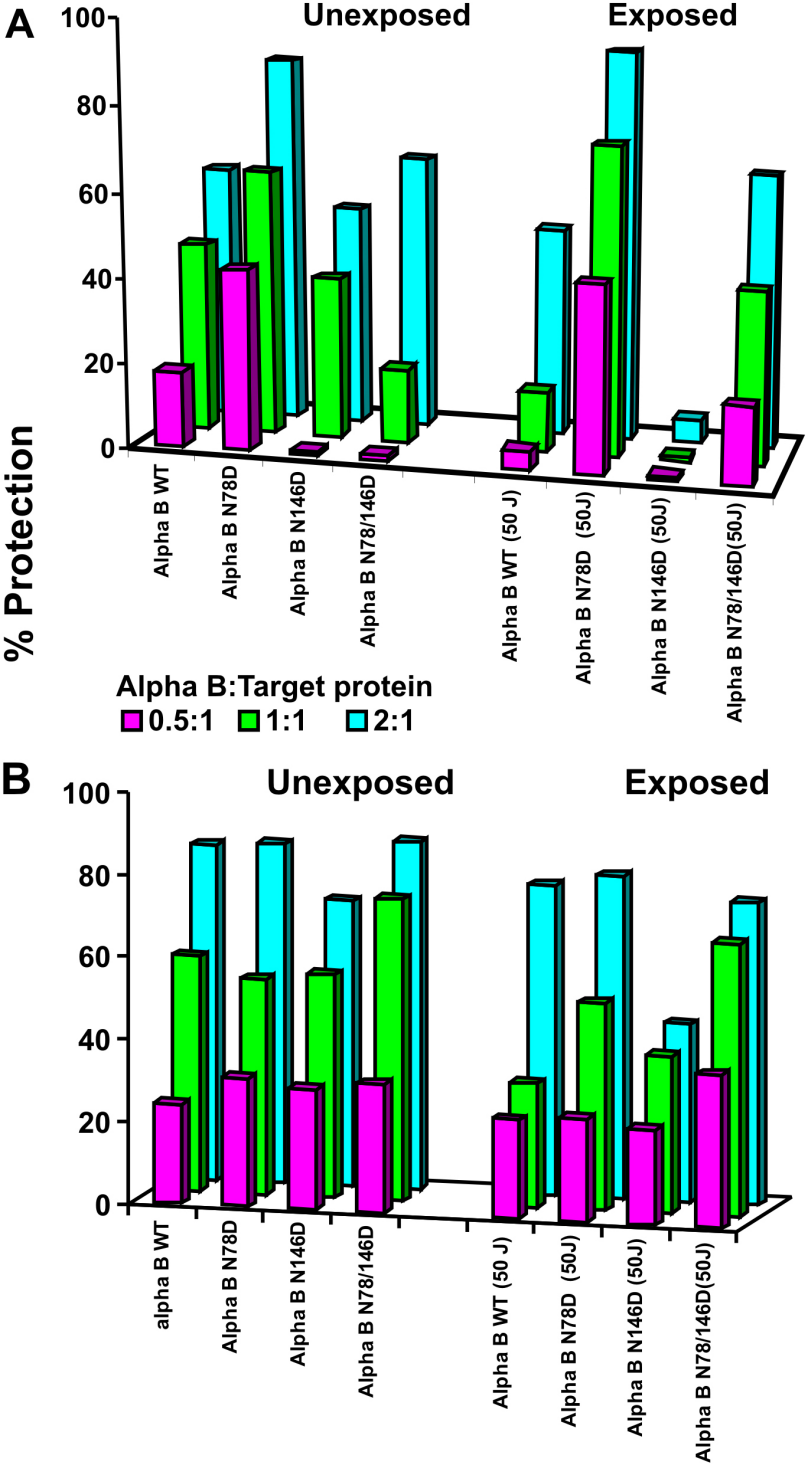
Effects of UV-A-exposure (at 50 J/cm^2^) on chaperone activity of WT αB-crystallin and its three deamidated mutant proteins. The chaperone activities of WT αB-crystallin and the three deamidated species were determined using insulin or citrate synthase as the substrates. **A:** Shows the aggregation of insulin as a target protein in the presence of dithiothreitol at room temperature. The aggregation was monitored at 360 nm (due to light scattering) as a function of time at 25 °C. The aggregation of insulin (100 μg in 10 mM phosphate buffer, pH 7.4, containing 100 mM NaCl) was initiated by 20 mM DTT at varying chaperone-to-target protein ratios. **B:** Aggregation of citrate synthase at 43 °C in presence of varying concentrations of αB-crystallin. During the thermal aggregation assay, 100 μg of citrate synthase (in 50 mM phosphate buffer, pH 7.8, containing 150 mM NaCl and 2 mM EDTA) was incubated at 43 °C with various concentrations of αB-crystallin to obtain the desired chaperone-to-target protein molar ratios.

### Site-specific mutagenesis

Recombinant human WT αB-crystallin or its deamidated constructs, previously generated by Gupta and Srivastava [32], were used in this study. The deamidation of Asn to Asp residue at positions 78, 146, or both in αB-crystallin cDNA was introduced by using QuickChange site-directed mutagenesis kit and following the manufacturer’s instructions (Stratgene, La Jolla, CA). Briefly, 25 ng of αB-crystallin cDNA template was used, and the polymerase chain reaction (PCR) conditions were as follows: pre-denaturing at 95 °C for 30 s followed by 16 cycles of denaturing at 95 °C for 30 s, annealing at 55 °C for 1 min, and extension at 68 °C for 5 min. After digestion with *Dp*nI, the PCR product was transformed to XL1-Blue supercompetent cells. DNA sequencing at the University of Alabama at Birmingham Core Facility identified the positive clones.

### Expression and purification of wild-type and deamidated αB-crystallin mutant proteins

*E. coli* BL21 (DE3) pLysS was transformed with mutant amplicons using a standard *E. coli* transformation procedure. The proteins were overexpressed with the addition of isopropyl-beta-D-thiogalactopyranoside (final concentration of 1 mM), and the cultures were incubated at 37 °C for 4 h. Bacterial cells, collected by centrifugation, were lysed in 50 mM Tris-HCl buffer, pH 7.5, containing 0.5 mM EDTA and 0.3 mM NaCl (TEN buffer). The isolation and purification of the WT and mutant proteins were performed as described earlier by Gupta and Srivastava [32]. Briefly, the soluble fraction was dialyzed against 50 mM Tris-HCl, pH 7.9, containing 0.5 mM EDTA and 1 mM dithiothreitol (TED buffer), and subjected to diethylaminoethyl-Sephacel ion-exchange chromatography (2.5×35 cm column). The bound proteins were eluted with a gradient of 0-0.5 M NaCl in TED buffer (flow rate 0.5 ml/min, total volume 120 ml). The fractions, which contained WT αB-crystallin or its mutant proteins and identified by sodium dodecyl sulfate-polyacrylamide gel electrophoresis (SDS–PAGE), were pooled and subjected to hydrophobic chromatography using a Butyl-Sepharose column. The column (2.5×30 cm) was equilibrated with 50 mM phosphate buffer, pH 7.0, containing 0.5 M (NH_4_)_2_SO_4_, and the resin-bound proteins were eluted with a decreasing (NH_4_)_2_SO_4_ concentration (i.e., 0.5 M to 0 M, total volume 500 ml). The fractions, which contained αB-crystallin and were identified by SDS–PAGE, were concentrated by either lyophilization or ultrafiltration (using a 10,000 kDa molecular weight cut off Amicon membrane under N_2_). The minor contaminating bands from the WT and mutant protein preparations were removed by size-exclusion HPLC using a TSK G-3000PW_XL_ column. The samples were eluted with 50 mM sodium phosphate buffer, pH 7.5. The purity of WT and mutant αB-crystallin species was examined by SDS–PAGE and two-dimensional (2D)-gel electrophoresis. The concentrations of the proteins were determined using the Pierce protein determination kit or by their absorbance at 280 nm.

**Figure 3:**
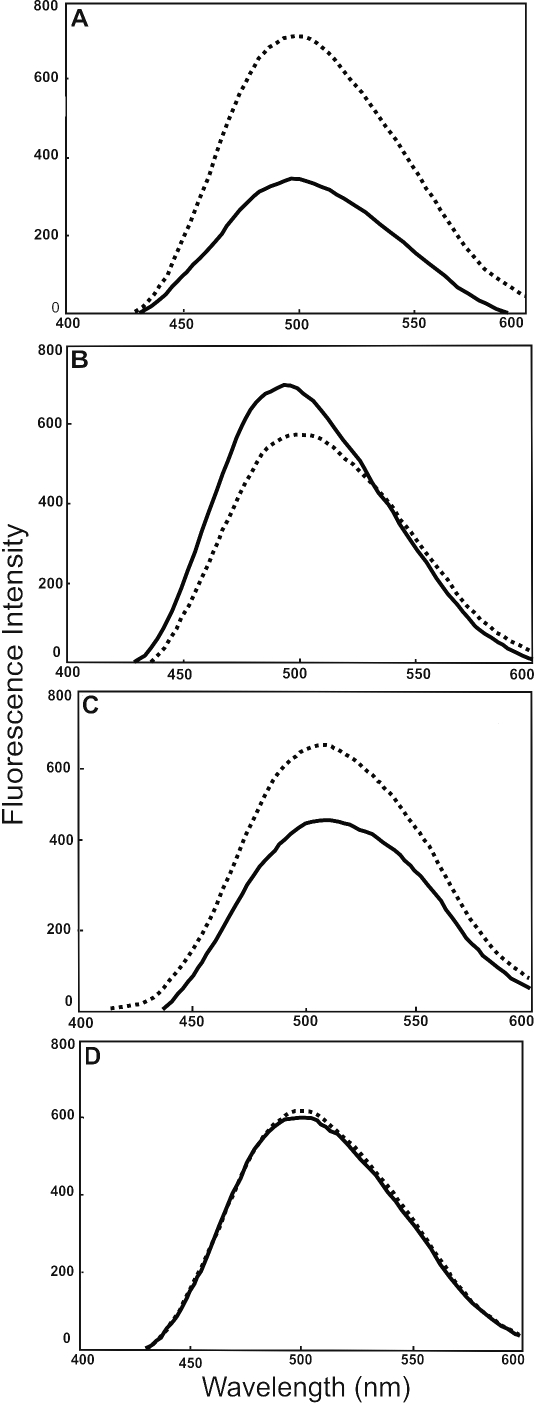
ANS-binding and fluorescence determination of UV-A-exposed and UV-A-unexposed WT- and deamidated αB-crystallin species. Fluorescence spectra of UV-A-unexposed (^____^) and UV-A-exposed (-------; at 50 J/cm^2^) of WT αB-crystallin and its three deamidated mutants after ANS binding. Fluorescence spectra following ANS-binding were determined as described in Methods. (**A**) WT αB-crystallin, (**B**) αB-Asn78Asp mutant, (**C**) αB-Asn146Asp mutant, and (**D**) αB-Asn78/146Asp mutant.

### Two-dimensional gel electrophoresis

The protein samples were mixed with resolubilization buffer [42], composed of 5 M urea, 2 M thiourea, 2% 3-[C3-cholamidopropyl]dimethyl-ammonio-1-propansulfonate (CHAPS), 2% caprylylsulfobetaine 3–10, 2 mM tri-butyl phosphine, and 40 mM Tris, pH 8.0, in the ratio 2:1 (protein: resolubilization buffer). Each sample was subjected to 2-D gel electrophoresis (isoelectric focusing in the first dimension and SDS–PAGE in the second dimension). Isoelectric focusing (IEF) separation was performed using Immobiline Dry Strips (pH range of 3–10) by following the manufacturer’s instructions (GE Biosceinces, Piscataway, NJ). SDS–PAGE in the second dimension was performed using a 15% polyacrylamide gel by Laemmli’s method [43]. The Ettan DALTsix Electrophoresis System (GE Biosciences) was used during SDS–PAGE.

**Figure 4:**
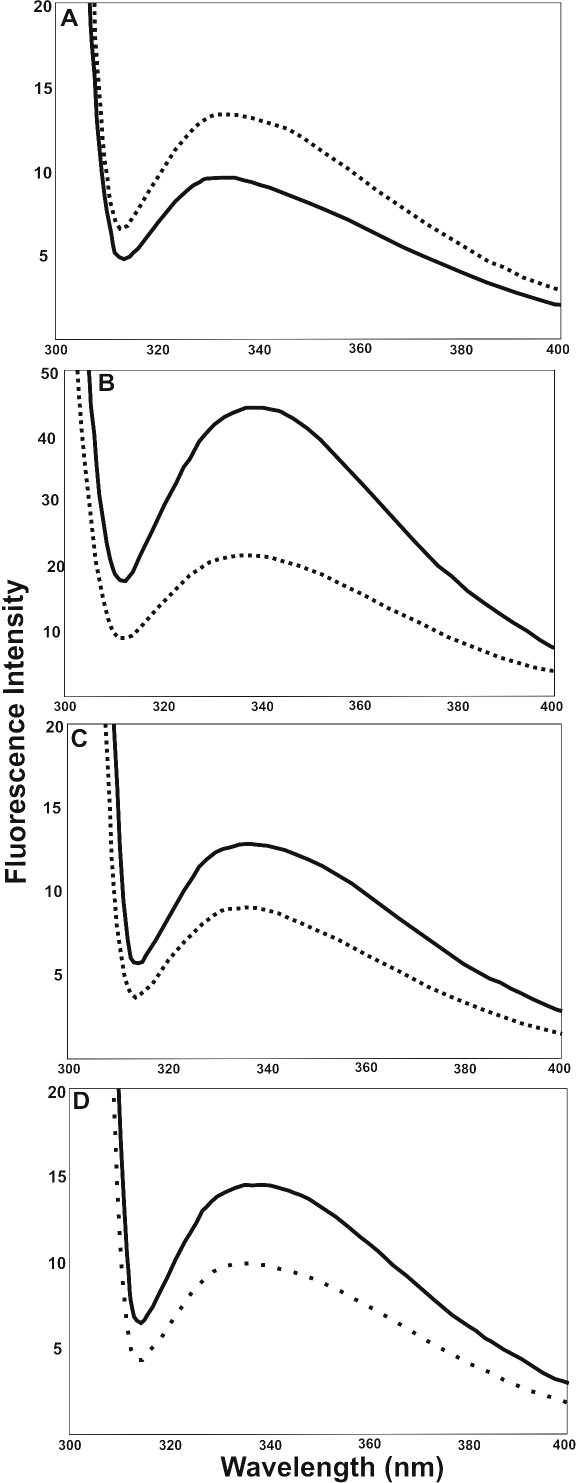
Intrinsic Trp fluorescence determination of UV-A-exposed and UV-A-unexposed WT- and deamidated αB-crystallin species. Intrinsic Trp fluorescence spectra of UV-A-unexposed (^____^) and UV-exposed (-----) WT αB-crystallin and its three deamidated mutants. Fluorescence spectra were determined as described in Methods. (**A**) WT αB-crystallin, (**B**) αB-Asn78Asp mutant, (**C**) αB-Asn146Asp mutant, and (**D**) αB-Asn78/146Asp mutant.

### UV-A-exposure of WT and deamidated αB-crystallin

The WT αB-crystallin and its three deamidated species were exposed to UV-A light (320–400 nm) using a Daavlin Research Irradiation Unit (Bryan, OH). The exposure doses were controlled using two Daavlin Flex Control Integrating Dosimeters. The instrument was equipped with an electronic controller to regulate UV-A dosage at a fixed distance of 22 cm from lamps to the surface of the vials containing the protein preparations. Individual protein preparations (in 50 mM phosphate buffer, pH 7.5) were exposed in open PCR tubes (200 μl in each tube) at intensities of 5, 20, or 50 J/cm^2^. The samples were exposed at a rate of 3 J/min and kept on ice to maintain the temperature at about 5 °C throughout the exposure.

**Figure 5:**
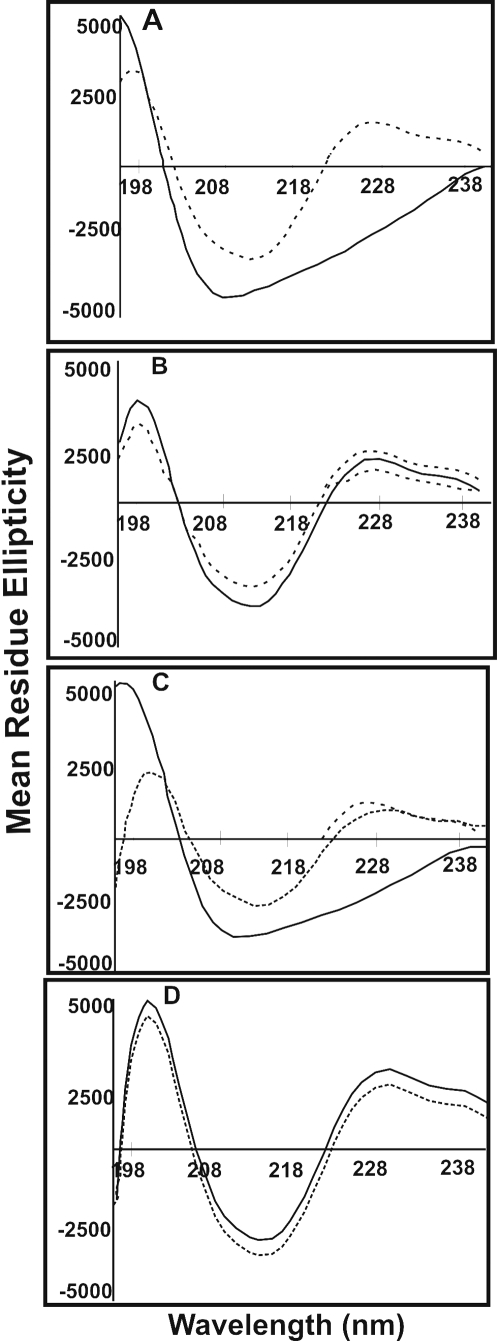
Determination of secondary structure by Far-UV circular dichroism spectroscopy of UV-A-exposed and UV-A-unexposed WT- and deamidated αB-crystallin species. Far-UV CD spectra of UV-A-unexposed (^____^) and UV-exposed (----) WT αB-crystallin and its three deamidated mutants. Far-UV CD spectra were determined as described in Methods. (**A**) WT αB-crystallin, (**B**) αB-Asn78Asp mutant (**C**) αB-Asn146Asp mutant, and (**D**) αB-Asn78/146Asp mutant.

### Chaperone activity assay of WT αB-crystallin and deamidated mutant proteins

The chaperone activities of WT αB-crystallin and the three deamidated species were determined using insulin or citrate synthase as the target proteins by the methods previously described [32]. During the chaperone assay, the aggregation of the target protein was induced either by thermal or non-thermal means. In these analyses, the aggregation of the target proteins was monitored at 360 nm (due to light-scattering) as a function of time (60 min) using a scanning spectrophotometer (model UV2101 PC; Shimadzu, Columbia, MD) equipped with a six-cell positioner (model CPS-260; Shimadzu) and a temperature controller (model CPS 260; Shimadzu). The aggregation of insulin as a target protein in the presence of dithiothreitol was monitored at 360 nm (due to light scattering) as a function of time at 25 °C. The aggregation of insulin (100 μg in 10 mM phosphate buffer, pH 7.4, containing 100 mM NaCl) was initiated by 20 mM DTT at varying chaperone-to-target protein ratios. During the thermal aggregation assay, 100 μg citrate synthase (in 50 mM phosphate buffer, pH 7.8, containing 150 mM NaCl and 2 mM EDTA) was incubated at 43 °C with various concentrations of αB species to obtain the desired chaperone-to-target protein molar ratios.

### MALDI-TOF and ES-MS/MS analysis

Matrix assisted laser desorption ionization-time of flight mass spectrometry (MALDI-TOF MS) analysis and electrospray tandem mass spectrometry (ES-MS/MS) sequencing (Micromass QTOF-2) were performed at the Comprehensive Cancer Center Mass Spectrometry Shared Facility of the University of Alabama at Birmingham. For mass spectrometric analysis, the individual protein spots were excised from a SDS–PAGE gel using pipette microtips. The polyacrylamide pieces containing individual bands were destained with three consecutive washes with a 50:50 mixture of 25 mM ammonium bicarbonate and acetonitrile for 30 min. Next, the samples were washed for 10 min with 25 mM ammonium bicarbonate before digestion with trypsin (12 ng/μl; sequencing grade from Roche, Pleasanton, CA) for 16 h at 37 °C. Peptide solutions were then extracted using 100 μl of a 50:50 mixture of 5% formic acid and acetonitrile for 30 min. Supernatants were collected and evaporated to dryness in a Savant SpeedVac (Thermoscientific, Waltham, MA). Samples were resuspended in 10 μl of 0.1% formic acid. C-18 ZipTips (Millipore, Billerica, MA) were used to desalt peptide mixtures before applying samples to the MALDI-TOF 96 well spot gold-coated target plates. Bound peptides were eluted from the ZipTips with 10 μl methanol. They were mixed (1/10 dilution) with a saturated solution of α-cyano-4-hydroxycinnamic acid (CHCA) matrix in 50% aqueous acetonitrile. Samples were allowed to dry before performing MALDI-TOF MS using a Voyager DE-Pro (ABI, Foster City, CA) with a nitrogen laser (337 nm) operating in the positive mode. Spectra were the sum of 100 laser shots; they were then de-isotoped and analyzed using Voyager Explorer software, and peptide masses were submitted to the MASCOT search engine for protein identifications. The potential identity of the proteins was determined by using the NCBInr database. Tandem mass spectral analyses were performed with the Q-TOF 2 mass spectrometer (Waters-Micromass, Manchester, UK) using electrospray ionization to verify the identity of the proteins. The tryptic peptides used for the MALDI-TOF MS analysis were then analyzed by LC-MS/MS. Liquid chromatography was performed using a LC Packings Ultimate LC-Switchos microcolumn switching unit and Famos-autosampler (LC Packings, San Francisco, CA). The samples were concentrated on a 300 μm i.d. C_8_ reverse-phase precolumn at a flow rate of 10 μl/min with 0.1% formic acid and then flushed onto a 10 cm×75 μm i.d. C_8_ reverse-phase column at 200 μl/min with a gradient of 5%–100% acetonitrile in 0.1% formic acid for 30 min. The nanoelectrospray ionization interface was used to transfer the LC eluent into the mass spectrometer. The Q-TOF was operated in the automatic switching mode where multiple-charged ions were subjected to MS/MS if their intensities rose above six counts. Protein identification was performed by either the ProteinLynx Global Server software or by manual interpretation.

### Intrinsic Trp fluorescence determinations

The fluorescence spectra were recorded using a Shimadzu RF-5301PC spectrofluorometer with excitation and emission band passes set at 5 and 3 nm, respectively. The total Trp intensities (between 300 and 400 nm) of the unexposed and the exposed WT αB-crystallin and its three deamidated species (0.2 mg/ml) in 50 mM phosphate buffer, pH 7.5, were recorded by excitation at 295 nm.

### 8-anilino-1-naphthalenesulfonate-binding

The binding of a hydrophobic probe, 8-anilino-1-naphthalenesulfonate (ANS), to unexposed and exposed WT αB-crystallin and deamidated mutant proteins was determined. ANS is nonfluorescent in aqueous solutions but becomes fluorescent when bound to hydrophobic patches on the surface of a protein. Therefore, it acts as a probe to determine changes in surface hydrophobic patches in a protein. Individual proteins (0.2 mg/ml) in 50 mM phosphate buffer, pH 7.5, containing 100 mM NaCl and ANS solution (final concentration 10 μM), were incubated for 15 min at 37 °C. The samples were left at room temperature for 5 min, and their fluorescence spectra were recorded between 400 and 600 nm by excitation at 390 nm.

### Circular dichroism spectroscopy

Far UV CD spectra of the WT αB-crystallin and deamidated species were recorded using an Aviv CD spectropolarimeter (Model 62 DS; JASCO, Easton, MD). Experiments were performed using 0.2 mg/ml of protein (filtered through 0.2 μm filter) in 50 mM potassium phosphate buffer, pH 7.5, using a 0.01-cm path length cell. Data were collected from 250 nm to 192 nm at 0.5 nm increments with 16 s averaging time per point. Data were baseline corrected, smoothed, and multiplied by a scaling factor to obtain spectra in units of mean residue ellipticity. Secondary structure calculations were performed using the SELICON program.

**Table 1:** The level (percentages) of secondary structure contents in WT αB and its three deamidated mutants.

Secondary structure contents	WT (UE)	WT (E)	Asn78Asp (UE)	Asn78Asp (E)	Asn146Asp (UE)	Asn146Asp (E)	Asn78/146Asp(UE)	Asn78/146 Asp(E)
α-Helix	27	3	5	3	29	3	3	2
β-Sheet	42	69	58	70	45	70	70	69
β-Turn	18	16	12	15	16	16	16	17
Random Coil	13	12	25	12	10	12	10	12

**Figure 6:**
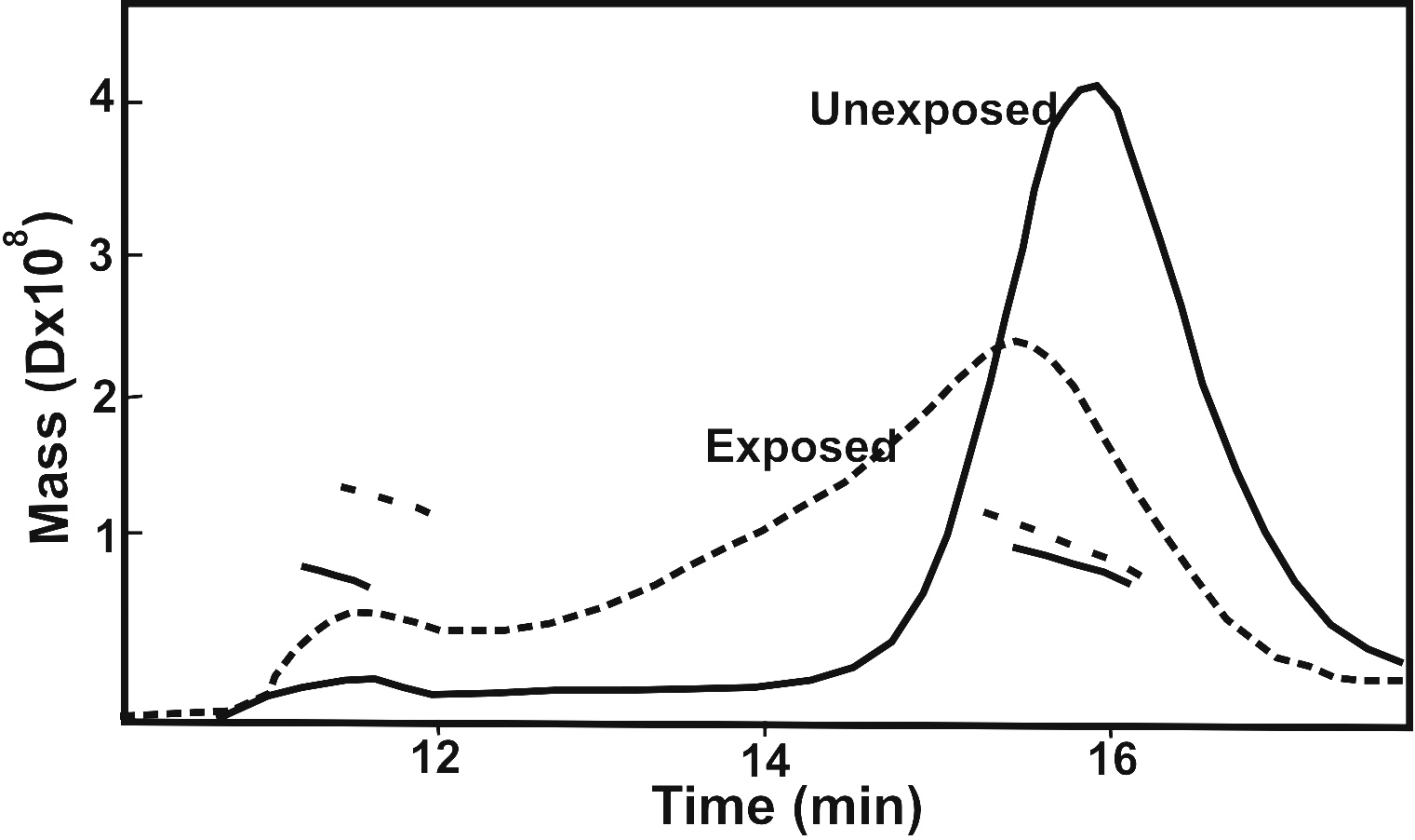
Determination of molecular mass by dynamic light scattering method of UV-A-unexposed and exposed WT αB-crystallin and its three deamidated mutants. A multiage laser light scattering instrument (Wyatt Technology, Santa Barbara, CA) coupled to a HPLC was used to determine the molecular mass of the WT protein and its deamidated mutant proteins. The figure shows elution profiles at 280 nm of UV-A-unexposed (^____^) and UV-A exposed (-----) αB-Asn146Asp mutant protein from a TSK G-5000PW_XL_ column as well as the molecular mass. Similar to the UV-A-exposed αB-Asn146Asp mutant protein profile, a high molecular weight (HMW) protein peak that eluted first (at about 10 min) followed by a crystallin peak (at about 15 min) were also observed for WT αB-crystallin and the αB-Asn78Asp and αB-Asn78/146Asp mutant proteins.

The secondary structure contents of the protein species were determined from the far UV-CD spectra as shown in Figure 6A. UE: Unexposed; E: UV-A-Exposed

### Molecular mass determination by dynamic light scattering

A multiangle laser light scattering instrument (Wyatt Technology, Santa Barbara, CA) coupled to HPLC with a TSK G-5000 PW_XL_ column (Montgomeryville, PA) was used to determine the absolute molar mass of the UV-A-unexposed and exposed WT αB-crystallin and its three deamidated proteins. Briefly, protein samples in 50 mM sodium phosphate, pH 7.4, were filtered through a 0.2 μm filter before their analysis. Results used 18 different angles, and the angles were normalized with the 90° detector.

## Results

### Confirmation of site-specific mutations in αB-crystallin mutants

Human lens αB-crystallin contains two Asn residues at positions 78 and 146. By using a point mutagenesis method, each of the two Asn residues was changed to Asp in two mutants (i.e., αB-Asn78Asp and αB-Asn146Asp mutants), and both Asn residues were changed to Asp residues in a double mutant protein (i.e., αBAsn78/146Asp). As described previously [32], DNA sequencing confirmed these mutations at the desired positions in the respective mutants. Additionally, specific mutations of Asn to Asp residues were also confirmed in the αB-Asn78Asp, αB-Asn146Asp, and αB-Asn78/146Asp mutants by matrix-assisted desorption ionization–time of flight (MALDI-TOF) mass spectrometry as described previously [32].

### Expression and purification of human recombinant αB-crystallin and its deamidated mutant proteins

The WT αB-crystallin and its three deamidated species were expressed in *E. coli*. The SDS–PAGE and MALDI-TOF analyses showed the expression of full-length recombinant αB-crystallin in the system used. The expressed proteins were purified to homogeneity by three consecutive chromatographic steps as described in the Methods. SDS–PAGE analysis showed a single protein band of about 20,000 Da in the purified preparation of WT αB-crystallin and in the single bands of the three purified deamidated mutant proteins (Figure 1).

### Comparative effects of UV-A-irradiation on WT αB-crystallin and its deamidated mutant proteins

When WT αB-crystallin and its three deamidated mutant proteins (at identical protein concentrations) were exposed to UV-A-irradiation at doses of 20 and 50 J/cm^2^, each protein showed increasing dimerization to a 40 kDa species and partial degradation with increasing UV-A-doses (Figure 1). In general, the degradation was greater in the three mutant proteins than in the WT αB-crystallin but was most pronounced in the αB-Asn146Asp mutant protein. Because of the treatment of samples with 150 mM β-mercaptoethanol before their analysis by SDS–PAGE, it is unlikely that the dimers were formed via non-disulfide bonding

### Comparative effects of UV-A-irradiation on chaperone activity of WT and deamidated αB-crystallin mutants

As stated above, the chaperone activity assay was performed by following DTT-induced aggregation of insulin at 25 °C or heat-induced (43 °C) aggregation of citrate synthase in either the absence or presence of WT αB-crystallin or three individual deamidated mutant proteins. Figure 2A shows chaperone activity as percent protection of DTT-induced aggregation of UV-A-irradiated (at 50 J/cm^2^) WT αB-crystallin and its three deamidated mutant proteins at three different ratios of αB-crystallin and insulin (i.e., 0.5:1, 1:1, and 2:1, αB-crystallin:insulin). At 0.5:1 ratio, the UV-A non-irradiated WT αB-crystallin showed 20% protection and increased to about 65% at 2:1 ratio. In contrast, the UV-A-irradiated WT αB-crystallin showed 5% protection at 0.5:1 and 50% at 2:1 ratio, indicating a reduction in chaperone activity after UV-A-exposure. The non-irradiated αB-Asn78Asp showed about 45% protection at 0.5:1 ratio and 90% at 1:2 ratio, and the percent protection remained almost at the same levels even after UV-A-exposure, suggesting no effect of UV-A-exposure on the mutant protein. The non-irradiated αB-Asn146Asp and αB-Asn78/146Asp mutant proteins at 0.5:1 ratio exhibited almost no protection but increased to 55% and 65%, respectively, at 2:1 ratio. In contrast, on UV-A-exposure, the αB-Asn146Asp mutant protein showed no protection at both 0.5:1 and 2:1 ratios, whereas the UV-A-exposure chaperone activity (i.e., percent protection) was not altered in αB-Asn78/146Asp protein.

On determination of citrate synthase-denaturation at 43 °C, the UV-A-exposed αB-Asn146Asp mutant showed maximum loss in chaperone activity compared to either WTαB-crystallin and αB-Asn78/146Asp and αB-Asn78/146Asp mutant proteins (Figure 2B). However, the loss in chaperone activity was about 50% compared to the WT αB-crystallin at 2:1 ratio (αB:citrate synthase). Therefore, at this highest ratio of αB-crystallin to target protein (insulin or citrate synthase), the loss of chaperone activity in the αBAsn146Asp mutant relative to other αB-crystallin species showed similar results. The gain in chaperone activity in αB-Asn146Asp protein with citrate synthase as the substrate at an elevated temperature compared to insulin at room temperature could be due to exposed hydrophobic patches.

**Table 2:** Molecular weights (in Dalton) of unexposed and UV-A-exposed WT αB and its three deamidated mutant proteins.

Protein	Type	Unexposed	UV-A exposed
WT αB	HMW	1.84×10^6^	4.61×10^7^
	Crystallin	4.28×10^5^	5.00×10^5^
			
Asn78Asp	HMW	None	1.60×10^6^
	Crystallin	5.30×10^5^	5.30×10^3^
			
Asn146Asp	HMW	None	2.6×10^7^
	Crystallin	4.20×10^5^	4.96×10^5^
			
Asn78/146Asp	HMW	6.60×10^5^	3.70×10^7^
	Crystallin	5.01×10^5^	6.23×10^5^

Oligomer size determination of UV-A-unexposed and UV-A-exposed WT αB-crystallin and its three deamidated proteins by a dynamic light scattering method. A multiangle-light scattering instrument coupled to a HPLC system was used to determine the absolute molecular molar mass. Results used 18 different angles, and the angles were normalized with 90^o^ detector.

The results suggested that compared to the deamidation of the Asn to Asp residue in αB at position 78 or at both positions 78 and 146, the single deamidation at position 146 position was lethal, resulting in a complete loss of chaperone activity.

### Structural changes in WT αB-crystallin and its three deamidated mutant proteins following UV-A-irradiation

To determine reasons for a complete loss in the chaperone activity in αB-Asn146Asp mutant on UV-A-exposure but not in αB-Asn78Asp and αB-Asn78/146Asp mutant proteins compared to WT αB-crystallin, the comparative structural changes in these proteins were investigated.

#### ANS binding

The changes on ANS binding in fluorescence spectra of WT αB-crystallin and its three deamidated mutant proteins before and after UV-A-irradiation (at 50 J/cm^2^) are shown in Figure 3. The UV-A-exposed WT αB-crystallin showed a threefold increase in fluorescence with a slight red shift from 498 to 500 nm compared the unexposed WT protein (Figure 3A), suggesting increased exposure of surface hydrophobic patches in the protein upon UV-A-exposure. In contrast to the WT αB-crystallin, the αB-Asn78Asp mutant protein upon UV-A-exposure showed a decrease in fluorescence with a red shift from 495 to 498 nm relative to its unexposed species (Figure 3B). The αB Asn146Asp protein exhibited an increase in fluorescence with a blue shift from 509 to 507 nm relative to the unexposed mutant protein (Figure 3C). However, the double mutant protein (αB-Asn78/146Asp) showed no increase in the fluorescence, although a blue shift from 501 to 498 was seen. A red shift and 501 to 498 nm on ANS binding in WT αB-crystallin and αB-Asn78Asp mutant after UV-A-exposure suggested greater exposure of their surface hydrophobic patches, whereas a blue shift in both αB-Asn146Asp and αB-Asn78/146Asp proteins suggested relatively buried surface hydrophobic patches. The unusual behavior of a blue shift and an increase in the fluorescence in αB-Asn146Asp mutant protein suggested a relatively compact structure with an increase in surface hydrophobic patches upon UV-A exposure.

**Figure 7:**
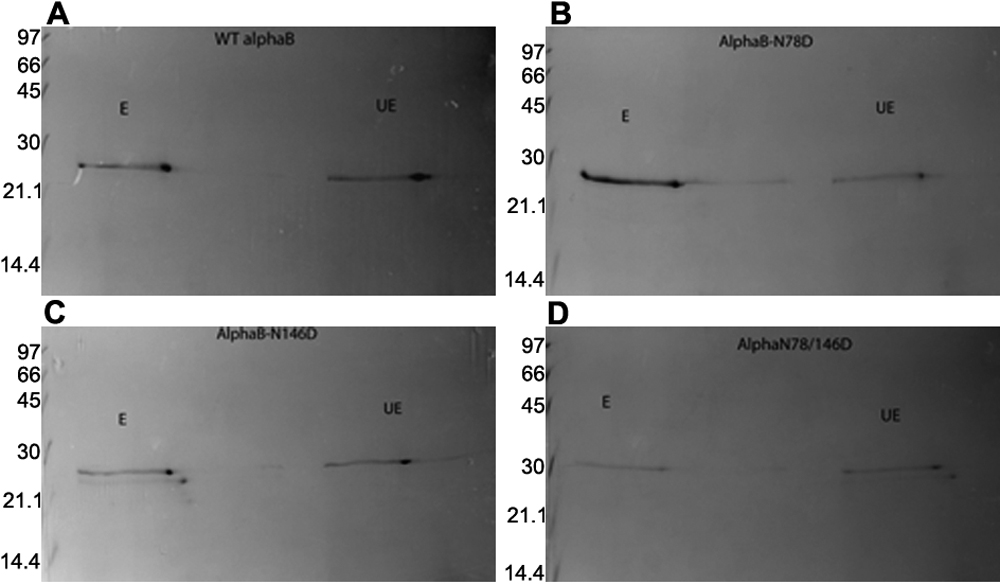
Two-dimensional protein profile of UV-A-exposed and unexposed WT αB and its three deamidated mutant proteins. **A-D** show UV-A-exposed (E) and UV-A unexposed (UE) 2D-gel electrophoretic profiles of WT αB-crystallin and its three deamidated mutant proteins. The unexposed and exposed preparations from each of the proteins were first separated by IEF in the first dimension and then by SDS–PAGE in the second dimension. Four spots from each profile were identified (see Figure 8) and were analyzed by the MALDI-TOF mass spectrometric method.

#### Intrinsic Trp fluorescence

Human lens αB-crystallin contains two Trp residues at positions 9 and 60, and their intrinsic fluorescence was determined in the UV-A-exposed and unexposed αB-crystallin-species. UV-A-exposed WT αB-crystallin showed a twofold increase in fluorescence with a blue shift of 3 nm with the λ_max_ increase from 332 to 335 nm compared to the unexposed control (Figure 4A). In contrast, the αB-Asn78Asp mutant protein on a similar exposure showed a decrease in its fluorescence intensity and a blue shift of 4–5 nm (339 nm versus 335 nm) relative to the unexposed control (Figure 4B). The αB-Asn146Asp protein also showed a decrease in fluorescence with a blue shift in λ_max_ from 338 nm to 333 nm relative to the unexposed control. After the UV-A-exposure of the αB-Asn78/146Asp mutant protein, a decrease in fluorescence intensity with a blue shift was seen compared to the unexposed control. Together the data showed varied changes in the Trp microenvironment of the deamidated mutant proteins after UV-A exposure, suggesting that the exposure resulted in an altered structure of the deamidated proteins relative to their unexposed respective proteins and also WT αB-crystallin.

#### Far-UV circular dichroism spectra

The far-UV circular dichroism (CD) spectra of the UV-A-exposed and unexposed WT αB-crystallin and its three deamidated mutant proteins were determined to gain information regarding their secondary structural changes (Figure 5). The CD spectra of each species in Figure 5 were baseline corrected, smoothed, and multiplied by a scaling factor to obtain spectra in units of mean residue ellipticity. Secondary structure calculations were performed with the SELCON program (Table 1) to find the percentages of α-helices, β-sheets, β-turns, and random coils. Because WT αB-crystallin has mostly β-sheet structures (42%), the changes in the β-sheet contents of each species before and after UV-A-exposure were of particular interest. The WT αB-crystallin showed an increase in β-sheet content from 42% to 69% upon UV-A-exposure. Compared to the WT αB-crystallin, the αB-Asn78Asp and αB-Asn146Asp mutant proteins also showed increases in β-sheet content from 58% to 70% and 45% to 70%, respectively, but the αB-Asn78/146Asp showed almost no increase (70% to 69%). These results were also evident from the ellipticity of these proteins (Figure 5). The α-helix contents of the three mutant proteins (αB-Asn78Asp, αB-Asn146Asp, and αB-Asn78/146Asp) decreased compared to the WT αB-crystallin upon UV-A-exposure, but the decrease was most evident in WT αB-crystallin and αB-Asn146Asp mutants. Similarly, the β-turn contents were also slightly lower in the three αB-crystallin mutants relative to WT αB-crystallin, and these values were unaffected by UV-A-exposure. Compared to WT αB-crystallin, the random coil structure showed maximum changes in the αB-Asn78Asp mutant protein while little changes in its levels were seen in the αB-Asn146Asp and αB-Asn78/146Asp mutant proteins, and these values were significantly reduced in αB-Asn78Asp mutant while they were only slightly lower in αB-Asn146Asp and αB-Asn78/146Asp mutants (Table 1).

**Figure 8:**
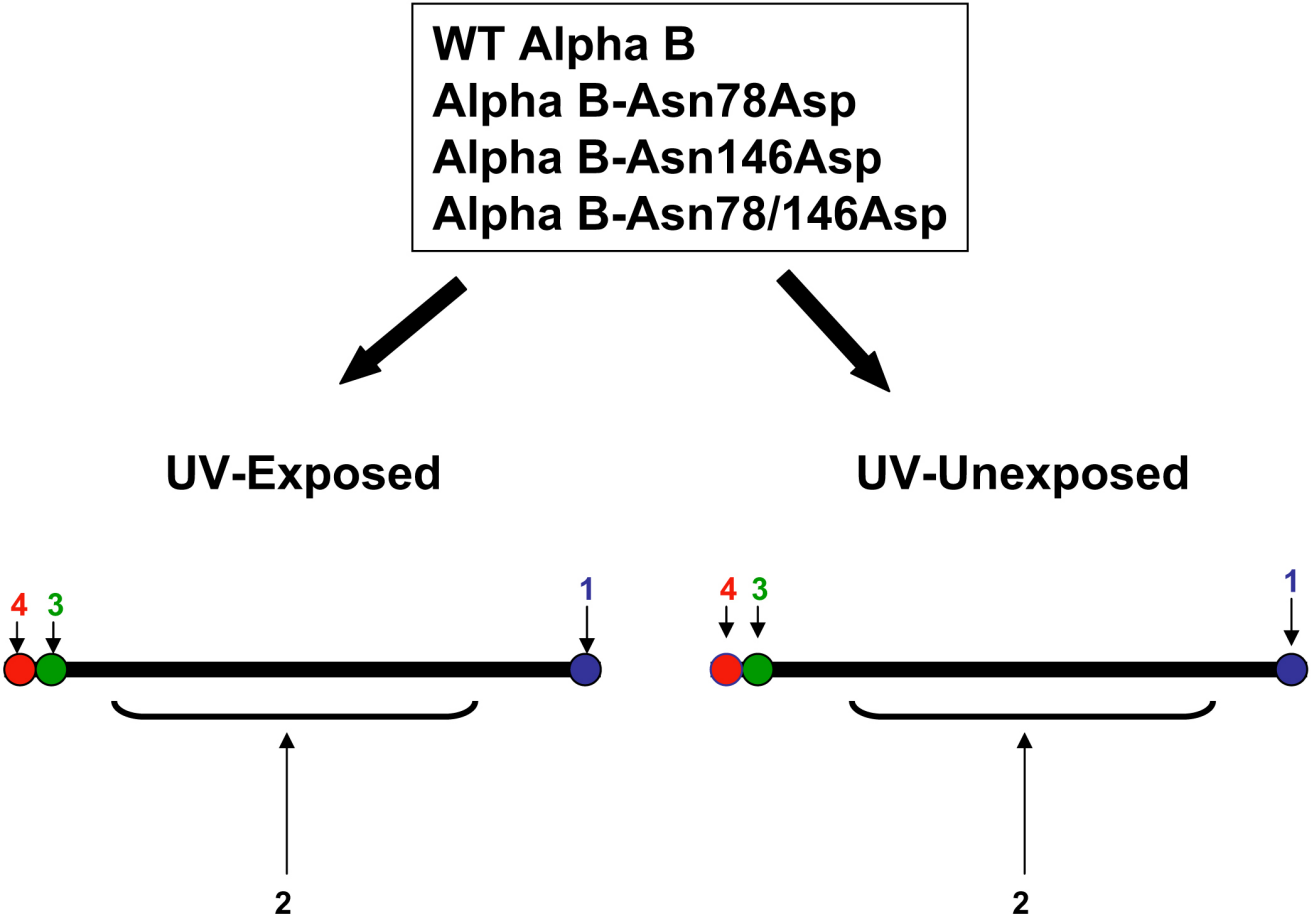
A schematic representing the four spots recovered from the two-dimensional gel profiles of the WT αB-crystallin and individual deamidated mutant proteins. These are numbered as spot number 1 to 4. For mass spectrometric analysis, the individual protein spots were excised from a SDS-PAGE gel. After destaining of the individual spots, the samples were washed for 10 min with 25 mM ammonium bicarbonate before digestion with trypsin (12 ng/μl) for 16 h at 37 °C. Peptide solutions were then extracted using 100 μl of a 50:50 mixture of 5% formic acid and acetonitrile for 30 min. Supernatants were collected and evaporated to dryness. Samples were resuspended in 10 μl of 0.1% formic acid and desalted using C-18 ZipTips. The samples were spotted to the MALDI-TOF 96 well gold-coated target plates after mixing with cyano-4-hydroxycinnamic acid (CHCA) matrix. MALDI-TOF MS was performed, and spectra were collected. The identity of the proteins was determined by using the NCBI database.

#### Determination of molecular mass by dynamic light scattering

As described in Methods, a multiangle laser light scattering instrument (Wyatt Technology, Santa Barbara, CA) coupled to HPLC with TSK G-5000 PW_XL_ column (TosoHaas, Montgomeryville, PA) was used to determine the absolute molecular mass of the UV-A-exposed and unexposed WT αB-crystallin and its three deamidated proteins. The typical elution profile from the TSK G-5000 PW_XL_ column at 280 nm of αB-Asn146Asp mutant protein preparation before and after UV-A exposure is shown in Figure 6. Following UV-A-exposure, a high molecular weight (HMW) protein peak typically eluted first (at about 10 min) followed by the crystallin peak (at about 15 min; Figure 6). The unexposed WT αB-crystallin showed a mass 4.28×10^5^ Da for the crystallin peak and 1.84×10^6^ Da for the HMW protein peak. The HMW protein peak, although present in WT and αB-Asn78/146Asp but absent in αB-Asn78Asp and αB-Asn146Asp, appeared in all proteins after UV-A-exposure. On UV-A exposure, the molecular mass of WT protein was increased, 5×10^5^ Da for the crystallin peak and 4.6×10^7^ Da for the HMW peak (Table 2). The αB-Asn78Asp mutant protein showed a mass of 5.3×10^5^ Da, which after UV-A-exposure, produced a HMW protein peak with a mass of 1.6×10^6^ Da. The UV-A-exposure of αB-Asn146Asp mutant protein also produced a HMW protein with a molecular mass of 2.6×10^7^ Da while the αB-crystallin molecular mass increased from 4.2×10^5^ Da to 4.96×10^5^ Da after UV-A exposure. A similar increase in the molecular mass of αB-crystallin and HMW protein was observed in αB-Asn78/146Asp mutant protein, the αB-crystallin molecular mass increased from 5.01×10^5^ Da to 6.23×10^5^ Da and the mass of HMW protein from 6.6×10^5^ Da to 3.7×10^5^ Da. Together, the results showed that the UV-A-exposure resulted in the aggregation of WT and its deamidated mutant proteins with species of greater mass than their respective unexposed species. In spite of an increase in mass in the HMW protein in WT αB-crystallin and αB-Asn146Asp mutant protein, which ranged between 2.7×10^7^ Da to 4.6×10^7^ Da, only the mutant protein lost its chaperone activity.

#### Oxidative changes

**Figure 9:**
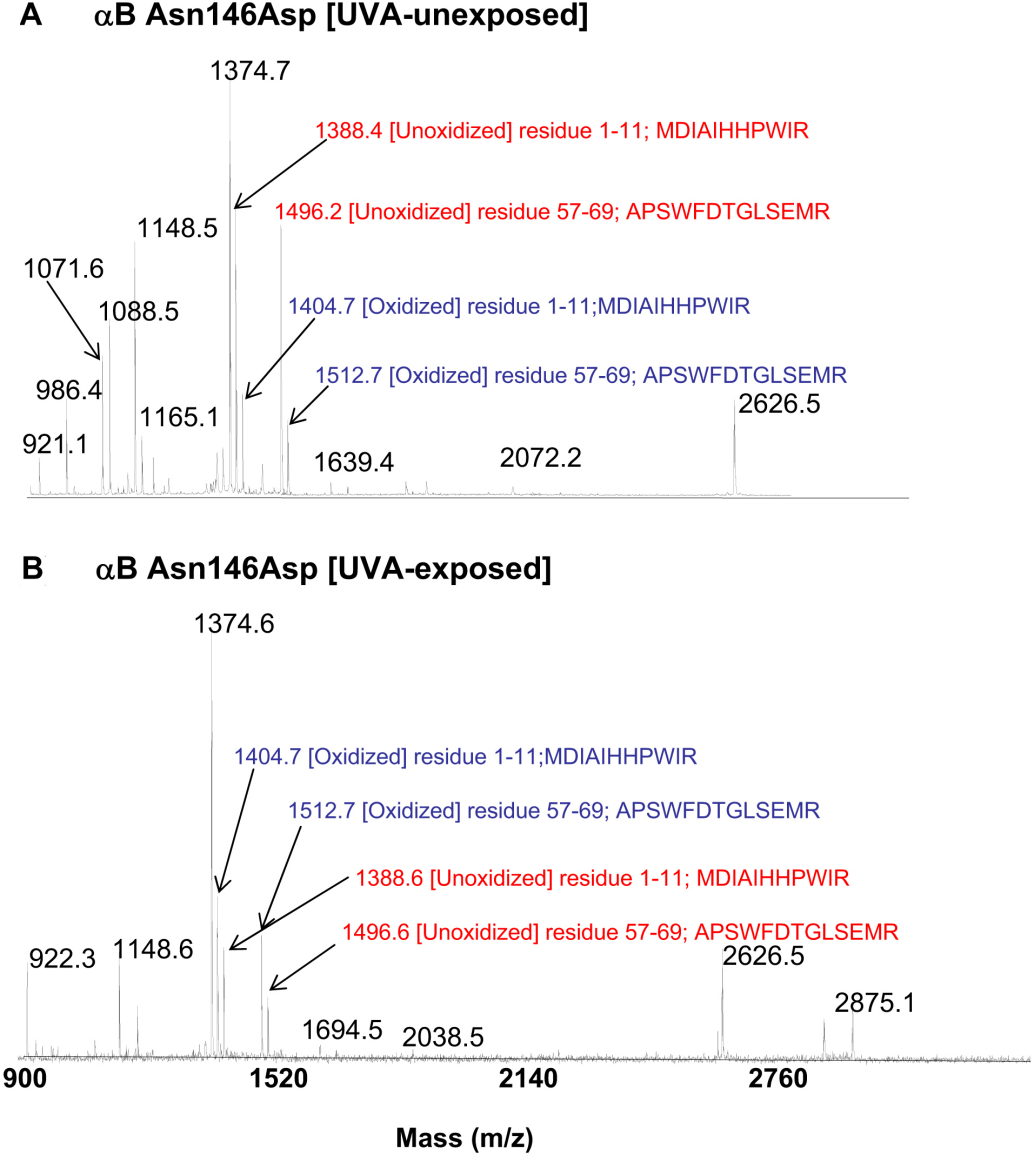
MALDI-TOF mass spectrometric tryptic profiles of spot 1 in the UV-A-exposed and unexposed αB-Asn146Asp mutant protein. The MALDI-TOF mass spectrometric tryptic profiles of UV-A-unexposed αB-Asn146Asp mutant (**A**) and UV-A-exposed αB-Asn146Asp mutant (**B**) is shown. The MALDI-TOF profiles of WT αB-crystallin and αB-Asn78Asp and Asn78/146Asp mutant proteins were similar to the profile of αB-Asn146Asp mutant protein as shown in Figure 9A and B. The profiles were used to determine oxidation of M and W residue peptide 1 with residues 1–11 [MDIAIHHPWIR, *m/z* 1388] and peptide 2 with residues 57–69 [APSWFDTGLSEMR, *m/z* 1496]. The ratio of peak heights of oxidized (shown in blue) versus unoxidized (shown in red) species of peptides 1 and 2 were calculated used, and the data are shown in Table 3.

Because the present literature suggests that oxidation of mainly Met and Trp residues readily occurs on UV-A-exposure (see Discussion), the oxidation of these residues in tryptic peptides of WT αB-crystallin and its three deamidated mutants were analyzed. The αB-crystallin contains two Met residues (at positions 1 and 68) and two Trp residues (at positions 9 and 60). Because both MALDI-TOF and ES-MS/MS analyses of the tryptic peptides from UV-A-exposed and unexposed WT αB-crystallin and its three deamidated mutants showed oxidation of Met and Trp residues, no conclusive results regarding their oxidation on UV-A exposure could be obtained. In these analyses, the UV-A-exposed and unexposed species that were recovered either after one-dimensional SDS–PAGE or present in a solution after the exposure was used. In an alternative approach, both UV-A-exposed and unexposed preparations of WT αB-crystallin and the deamidated mutant proteins were resolved by 2D-gel electrophoresis, which typically showed four spots (identified as spots 1, 2, 3, and 4 as shown in Figures 7 and 8). Each individual spot was excised, trypsin-digested, and examined by MALDI-TOF MS and ES-MS/MS methods. The focus in the analyses was the Met and Trp residues of two tryptic peptides, peptide 1 with residues 1–11 [MDIAIHHPWIR, *m/z* 1388] and peptide 2 with residues 57–69 [APSWFDTGLSEMR, *m/z* 1496]. Typical MALDI-TOF MS tryptic mass fragment profiles of spot no. 1 from UV-A-unexposed and exposed WT αB-crystallin are shown in Figure 9A and B. Similar profiles for spots 1, 2, 3, and 4 from the WT and its three deamidated proteins were also obtained. To obtain semi-quantitative data on the oxidation of Met and/or Trp residues during UV-A exposure, the relative differences in the ratios of peak heights between the oxidized tryptic peptide with *m/z* of 1404 (containing residue numbers 1–11) and the unoxidized peptide with *m/z* of 1388 were determined in the MALDI-TOF MS profiles of spot numbers 1-4. Similarly, the peak ratios were also determined from the MALDI-TOF MS profiles of peptide 2 (containing residue numbers 59-69) with *m/z* of 1512 (oxidized) and 1496 (unoxidized) for spots 1-4 for each αB-crystallin species. These ratios are shown in Table 3. A higher ratio of 1 or above in the UV-A-exposed versus unexposed species suggested that there was oxidation of the Met and/or Trp residues. Spot 1 in αB-Asn146Asp mutant was oxidized but not in the other two deamidated mutant proteins or in the WT αB-crystallin (Table 3). Together, the results suggested that the Met and/or Trp residues were oxidized by UV-A exposure in the WT αB-crystallin and αB-Asn146Asp but not in the αB-Asn78Asp and αB-Asn78/146Asp mutants. Furthermore, it is also suggested that certain specific oxidized species could only be resolved by 2D-gel electrophoresis to determine their oxidation by the MALDI-TOF MS method.

## Discussion

The purpose of this study was to determine the comparative effects of UV-A-irradiation on structural and functional properties of WT αB-crystallin and its three deamidated mutant proteins (αB-Asn78Asp, αB-Asn146Asp, and αB-Asn78/146Asp). We undertook this present study because the deamidation of crystallines in aging human lenses has been shown to be one of the most abundant posttranslational modifications [38] and UV-A-effects on structural and functional properties of deamidated αB-crystallin are presently unknown.

As stated above, the exposure to UV light has been shown to correlate with the development of cataract [8,19,44-51]. In some of these studies, latitude and sunlight exposure hours were positively correlated with cataract incidence. Although UV-B constitutes only solar 3% of UV radiation that reaches the earth, its effect varies with geographical, physical, and meteorological factors [51]. However, because UV-B light is absorbed by the cornea and aqueous humor of the human eye, most of the UV-A light reaches the lens and is absorbed by it [52,53]. The corneal epithelium and Bowman’s layer are the effective absorbers of UV-B radiation [53,54]. The in vivo action spectrum for UV-induced lens opacities in rabbits begins at 295 nm and extends to 320 nm [54]. Using a 6.6 nm full band pass, 300 nm radiation was found to be 30 times more effective than 315 nm radiation in producing lens opacities in vivo [54]. The UV radiation above 300 nm is partially transmitted by cornea, 2%–20% in the rat [53], 24% in rabbit [54], and 9% in human [55]. Therefore, UVB is a minor component of the total energy that reaches to the surface of the human lens. Nevertheless, old human lens proteins absorb two times more UV-A and visible light than UVB [55]. Taken together, these reports show that the cornea protects the lens from damaging UVB radiation, which is very significant in view of the increasing depletion of the ozone in the stratosphere, which is resulting in increased levels of UV radiation reaching the Earth.

The mechanism of UV-A-induced lens opacification is presently not fully understood. UV exposure of proteins from older human lens with a yellow color were seen to cause several reactive oxygen species to form [12,56,57]. Reports have suggested that UV-A-induced lenticular damage occurs because of the accumulation of several UV-A-absorbing compounds in the lens with aging and cataract, and these compounds act as UV-A-responsive sensitizers and produce reactive oxygen species, mostly ^1^O_2_ [56-58]. It has been reported that in young lenses, UV light that reaches the lens is absorbed by UV filters, such as either Trp residues [UVB] or 3-hydroxy-kynurenine glycoside O-β-D-glucoside (UV-A) and related compounds [58]. In the older lenses, the levels of the filters significantly decrease [58] with simultaneous increase in the UV-A-absorbing protein-bound chromophores. These chromophores act as sensitizers and generate reactive oxygen species. In young lenses, the UV filters that are produced due to catabolism of Trp, 3-hydroxykynurenine O-β-D-glucoside, 4-(2-amino-3-hydroxyphenyl)-4-oxabutanoic acid O-β-glucoiside, kynurenine, and 3-hydroxykynurenine [58-60], absorb UV-A light and thus protect the lens and retina from UV damage [59]. Lens coloration has been shown to occur due to the interaction of crystallines with a UV filter compound, 3-hydroxykynurenine, yielding an unsaturated ketone that is susceptible to a nucleophilic attack by cysteine, histidine, and lysine residues [59,60]. The modified amino acid containing proteins leads to lens coloration and might play a role in human nuclear cataractogenesis. Ortwerth et al. [20,56,57] have shown that these lens chromophores act as UV-A responsive sensitizers that produce reactive oxygen species (ROS), mostly as singlet oxygen, which in turn oxidizes Trp, His, and Cys residues within the lens proteins. Additional studies have suggested that UV-A-responsive sensitizers exist in aging human lenses and might be causing age-dependent lens protein modifications. Together, these reports suggest a major role of UV filter compounds in the generation of ROS that could cause oxidative damage to specific amino acids of crystallines.

An intriguing finding of our study was that after UV-A exposure at 50 J/cm^2^ of WT αB-crystallin and its three deamidated mutants, the αB-Asn146Asp mutant showed a maximum loss of chaperone activity, whereas the activity of WT αB-crystallin and the αB-Asn78Asp and αB-Asn78/146Asp mutants remained almost unaffected. In our previous report [41], the deamidation in Asn146 but not in Asn78 showed profound effects on structural and functional properties of human αB-crystallin. To determine the potential mechanism of the loss of chaperone activity in αB-Asn146Asp mutant protein, we determined biophysical properties of UV-A-exposed and unexposed WT αB and its three deamidated mutant proteins (αB-Asn78Asp, αB-Asn146Asp, and αB-Asn78/146Asp). The αB-Asn146Asp mutant protein exhibited an unusual behavior of a blue shift and an increase in the fluorescence upon ANS binding, suggesting a relatively compact structure but an increase in surface hydrophobic patches after UV-A exposure. However, in spite of increased hydrophobic patches, this mutant protein lost its chaperone activity upon UV-A-exposure. Although both αB-Asn78Asp and αB-Asn146Asp mutant proteins showed a decrease in Trp fluorescence with a blue shift in λ_max_ from 338 nm to 333 nm relative to unexposed controls, only the latter mutant protein lost chaperone activity. In contrast, WT αB-crystallin after UV-A-exposure showed a twofold increase in Trp fluorescence with a blue shift of 3 nm (a λ_max_ decrease from 335 nm to 332 nm) compared to the unexposed control, suggesting a UV-A-induced altered Trp microenvironment. However, the chaperone activity of the WT protein was relatively unaffected by the exposure compared to the αB-Asn146Asp mutant protein. On far-UV CD spectral determination, the random coil structure showed maximum changes in αB-Asn146Asp mutant protein in comparison to WT or the two other deamidated mutants. Furthermore, after UV-A-exposure, the β-sheet contents of WT αB-crystallin and the three mutants proteins (αB-Asn78Asp, αB-Asn146Asp, and αB-Asn78/146Asp) increased, but the β-turn and random coil contents were unaffected following UV-A-exposure. The α-helix contents showed the most dramatic decrease in WT αB-crystallin and αB-Asn146Asp mutant (27%–29% to 3%), whereas this was unaffected in the αB-Asn78Asp and αB-Asn78/146Asp mutant proteins. Taken together, the structural changes as suggested by the changes in the contents of the β-sheets and α-helices, the ANS binding, and intrinsic Trp spectra in αB-Asn146Asp mutant protein could account for the loss of chaperone activity. An increased oxidation of Met and Trp residues was also seen in Spot 1 of the UV-A-exposed αB-Asn146Asp mutant protein compared to its unexposed species, and the absence of such oxidation in the other two deamidated mutant proteins and WT αB-crystallin might also explain the loss in the chaperone activity.

Our results also suggest that there were UV-A-induced oxidative changes in Trp residue of WT αB-crystallin and its three deamidated mutants. Several previous reports have shown photo-destruction of Trp residues in purified crystallines after UV-exposure in the absence of any added sensitizers or UV filters. Schauerte and Gafni [61] showed in 1995 that UV-B exposure of bovine α-crystallin (obtained from Sigma Chemical Company) resulted in the decline of Trp fluorescence, suggesting the occurrence of modifications in these residues. Similarly, Fujii and Saito [62] showed a concomitant decrease in the fluorescence of Trp and an increase in N-formylkynurenine in purified bovine α-crystallin with increasing UV-C irradiation (254 nm). The UV-C exposed α-crystallin also showed an altered secondary structure, and the authors speculated that the changes were due to the UV absorption by the Trp residue that resulted in the photo-oxidation of Trp to N-formylkynurenine (NFK). NFK then acts as an efficient photosensitizer. They proposed that the excited triplet state of NFK reacts either with the ground state molecular oxygen to form ^1^O_2_ or the protein amino acids to produce free radicals. It has been shown in past studies that ^1^O_2_ and hydrogen peroxide can cause photo-damage to lens crystallines. We have also observed an UV-A-induced increase in NFK-fluorescence (excitation at 295 nm and emission at 330 nm) in WT αB-crystallin and its three deamidated mutant proteins (αB-Asn78Asp, αB-Asn146Asp, and αB-Asn78/146Asp; data not shown). Another report [63] showed that exposure of individual crystallines (bovine α-crystallin, βL-crystallin, recombinant βA3-crystallines, or γB-crystallines) to a 308 nm excimer laser resulted in each preparation exhibiting differential UV sensitivity in the form of light scattering, aggregation rate to dimers, and higher molecular weight species. A potential photo-oxidation mechanism, possibly involving Trp photoproducts (i.e., NFK), was speculated by the authors to account for the structural changes and aggregation of the crystallines. The exposure of the recombinant proteins to a 308 nm excimer laser was equivalent to irradiation of the proteins to a part of the UV-A-spectrum. The authors suggested that the effects on the recombinant proteins were possibly due to NFK that could act as a sensitizer during the UV exposure in the above described effects. We propose a similar mechanism for the UV-A irradiation effects on WT and the deamidated αB-crystallin.

**Table 3:** Determination of oxidation of methionine (M) and tryptophan (W) residues in peptide 1 (residues 1–11 [MDIAIHHPWIR, mass 1388]) and peptide 2 (residues 57–69 [APSWFDTGLSEMR, mass 1496]).

αB species		Spot 1	Spot 2	Spot 3	Spot 4
WT	Peptide 1	0.6	0.967	0.52	2.12
	Peptide 2	0.97	0.9	1.46	4.4
Asn78Asp	Peptide 1	25	0.005	0.05	0.85
	Peptide 2	0.87	0.001	0.003	0.94
Asn146Asp	Peptide 1	3.52	0.25	3.0	6.8
	Peptide 2	2.02	0.142	0.75	1.06
Asn78/146Asp	Peptide 1	10.8	2.0	1.24	ND
	Peptide 2	1.0	0.5	1.69	ND

The ratio of oxidized versus unoxidized species in peptide 1 and 2 of the 2D-gel separated as spot number 1 to 4 (Figure 9) were calculated. For peptide 1, the ratio of 1404:1388 and for the peptide 2, the ratio of 1512:1496 were calculated and shown in the table. ND means Not Determined.

Together, the above reports suggest that the Trp photo-oxidation products such as NFK could act as sensitizers during UV exposure. A similar potential mechanism might be operative during UV-A-exposure of the αB-crystallin species in the present study.

It was intriguing that the deamidation of Asn-146 resulted in a greater loss of chaperone activity compared to other αB-crystallin species used in this study. Based on similarities with the structures of other heat shock proteins (HSPs), it is believed that the NH_2_-terminal region (residue numbers 1–63 in αA-crystallin and numbers 1–66 in αB-crystallines) of α-crystallin forms an independently folded NH_2_-terminal domain, whereas the COOH-terminal region (residue numbers 143–173 in αA-crystallin and numbers 147–175 in αB-crystallin) is flexible and unstructured [6,64]. In addition, the middle conserved α-crystallin domain (residue numbers 64–142 in αA-crystallin and residue numbers 67–146 in αB-crystallin) plays a role in both substrate binding and chaperone activity [64]. Because Asn-146 belongs to the core region of the αB-crystallin domain and the importance of the core region of αB-crystallin in the chaperone activity is well established [65-67], the Asn146 might be involved in the three major factors (surface hydrophobic patches, subunit exchange rate, and hetero-oligomer sizes) that affect the chaperone activity in α-crystallin [68,69]. The hydrophobic patches seem to play an important role such as between human lens αA- and αB-crystallines; the residue numbers 42–57, 60–71, and 88–123 of αB-crystallin interacted with αA-crystallin [68]. Additionally, the β8 sequence (_131_LTITSSLS_138_) is an interactive domain in both αA- and αB-crystallines, and the surface exposed residues of β8 motif (Thr134, Ser 136, and Ser138) combined with surface-exposed residue of β3 motif (Asn78, Lys82, and His83) and the β9 motif (Gly141 and Thr14) form an interface for chaperone activity of αB-crystallin [66,67]. Because the Asn146 is adjacent to Gly141 in the conserved α-domain region of αB-crystallin, the resulting negative charge due to its deamidation (αB-Asn146Asp) might affect the β9 motif of αB-crystallin. This could explain the greater loss in the chaperone activity in the αB-Asn146Asp mutant protein. A missense mutation in the αB-crystallin (Arg120Gly) resulted in an inherited, adult onset desmin-related myopathy, a neuromuscular disorder where desmin (an intermediate filament protein) aggregated with αB-crystallin [70,71].

The above discussion presents the critical location of Asn146 residue within the core domain structure of αB-crystallin. Based on the results of the changes in biophysical properties of WT αB-crystallin and its three deamidated mutant proteins, no single structural change seem to explain the loss of the chaperone activity in the αB-Asn146Asp mutant protein. Apparently, the UV-A-induced oxidation and degradation of αB-Asn146Asp mutant protein show adverse effects on the chaperone activity of the mutant protein, which might be the most critical factor to explain the loss in the chaperone activity. Therefore, the effect of UV-A-induced oxidative insult is enhanced if the Asn146 residue is deamidated in αB-crystallin.
